# Metal Organic Frameworks Derived Fe-N-C Nanostructures as High-Performance Electrodes for Sodium Ion Batteries and Electromagnetic Interference (EMI) Shielding

**DOI:** 10.3390/molecules26041018

**Published:** 2021-02-15

**Authors:** Vadahanambi Sridhar, Inwon Lee, Hyun Park

**Affiliations:** 1 Global Core Research Centre for Ships and Offshore Plants (GCRC-SOP), Pusan National University, Busan 46241, Korea; sridhar@pusan.ac.kr (V.S.); inwon@pusan.ac.kr (I.L.); 2 Department of Naval Architecture and Ocean Engineering, Pusan National University, Busan 46241, Korea

**Keywords:** metal organic frameworks, microwave synthesis, sodium-ion batteries, EMI shielding

## Abstract

Metal organic framework (MOF)-derived carbon nanostructures (MDC) synthesized by either calcinations or carbonization or pyrolysis are emerging as attractive materials for a wide range of applications like batteries, super-capacitors, sensors, water treatment, etc. But the process of transformation of MOFs into MDCs is time-consuming, with reactions requiring inert atmospheres and reaction time typically running into hours. In this manuscript, we report the transformation of 1,4-diazabicyclo[2.2.2]octane, (DABCO)-based MOFs into iron nitride nanoparticles embedded in nitrogen-doped carbon nanotubes by simple, fast and facile microwave pyrolysis. By using graphene oxide and carbon fiber as microwave susceptible surfaces, three-dimensional nitrogen-doped carbon nanotubes vertically grown on reduced graphene oxide (MDNCNT@rGO) and carbon fibers (MDCNT@CF), respectively, were obtained, whose utility as anode material in sodium-ion batteries (MDNCNT@rGO) and for EMI (electromagnetic interference) shielding material (MDCNT@CF) is reported.

## 1. Introduction

Since the initial reports on electrochemical intercalation of lithium ions into graphite in 1979 [1], and commercialization of lithium ion batteries (LIB) by Sony in 1991 [2], lithium ion battery (LIB) are literally in our hands, powering laptop computers, tablets, cell phones, etc. But lithium is an extremely limited source; its distribution and accessibility is not uniform, with production dominated by the so-called ABC countries (Australia and Argentina, Bolivia and Brazil, Chile and China). As per some estimates, at the present consumption levels compounded with modest 5% annual increase, the mineable lithium resources can last for approximately 65 years [3]. Therefore, there is a growing necessity to pursue non- lithium batteries and researchers are actively investigating Na^+^ [4], K^+^ [5], Ca^2+^ [6], Mg^2+^ [7] etc.-based batteries. Amongst these, sodium ion batteries are gaining traction because sodium is the 6th most common element in earth’s crust, distributed widely and almost uniformly across countries and continents, have similar physical and electro-chemical properties to Li (except for larger the ionic radius of Na^+^ is 0.102 nm [8], which is larger than Li^+^ (0.076 nm)), which makes it a potential candidate to replace LIB, especially in static energy storage devices. However, the pioneering efforts by Dahn [9] and Komaba [10] showed that the larger size of Na^+^ and its sluggish reaction kinetics when compared to its lithium counterpart often results in very low capacity in carbon-based anodes.

One way of increasing the capacity of batteries is by using three-dimensional carbon nanostructured (3DCNS) electrodes. 3DCNS, due to their inherent mesoporous structure with high specific surface area, good mechanical integrity, and excellent electrical conductivity, are rapidly attracting immense attention from researchers especially in energy storage devices like batteries and supercapacitors. Syntheses of 3DCNS from metal organic framework (MOF) precursors by calcination/carbonization/pyrolysis has emerged as attractive technique and have been applied in energy storage [11], water treatment [12], photocatalysts [13], analysis of food [14], adsorbents for dye removal [15], polymerization catalysts [16], biosorbents [17], gas storage [18], etc. Besides, the functionality of MOF-derived carbons (MDC) can be augmented by incorporating hetero-atoms like sulfur, nitrogen, boron and nitrogen by choosing an appropriate functional organic linker. Despite having these advantages, the main drawback of MDC synthesis is since it involves prolonged calcination/carbonization/pyrolysis and annealing, which results in loss of porosity and surface area due to the destruction of the 3D framework (see Table S1 in supplementary file). One way of minimizing this loss in MDC porosity is by calcination/carbonization/pyrolysis of MOFs in conjunction with a substrate, which provides a substrate for MDC to anchor onto, thereby mitigating the agglomeration and aggregation of the metal nanoparticles by Ostwald ripening. Besides, suppose ‘functional’ substrates like graphene oxide or carbon nanotubes or carbon fibers are used. In that case, 3DCNS can be obtained, with excellent conductive pathways for rapid diffusion and distribution of electrolytes, which are critical in batteries and super-capacitors.

In this context, synthesis of 3DCNS from calcination and pyrolysis of MOF in conjunction with graphene [19], carbon fibers [20], nanoplatelets [21] etc. has been reported. Despite this progress, the major drawback of 3DCNS synthesis from MOF precursors is its prolonged synthesis time which in most cases runs into hundreds of minutes, requirement of high temperatures typically above 800 °C and the necessity of inert nitrogen or argon atmospheres. This prolonged pyrolysis time is not only time consuming but, in most cases, results in formation of highly agglomerated, low surface area metal nanoparticles embedded in a carbon matrix, which raises serious questions regarding the repeatability of synthesis of 3DCNS from MOF precursors. Therefore, there is a need for a fast and facile method to synthesize 3DCNS from MOFs.

In this manuscript, we report our newly developed strategy for rapid microwave synthesis of 3DCNS from MOF precursors. When graphene oxide and carbon fibers were used as microwave susceptible surfaces, three-dimensional MOF-derived nitrogen-doped carbon nanotubes anchored on reduced graphene oxide (MDNCNT@rGO) and carbon fibers (MDNCNT@CF) were obtained, respectively. The utility of MDCNT@rGO as anode material in sodium-ion batteries and MDNCNT@CF as EMI shielding material is reported.

## 2. Results and Discussion

**Figure 1:**
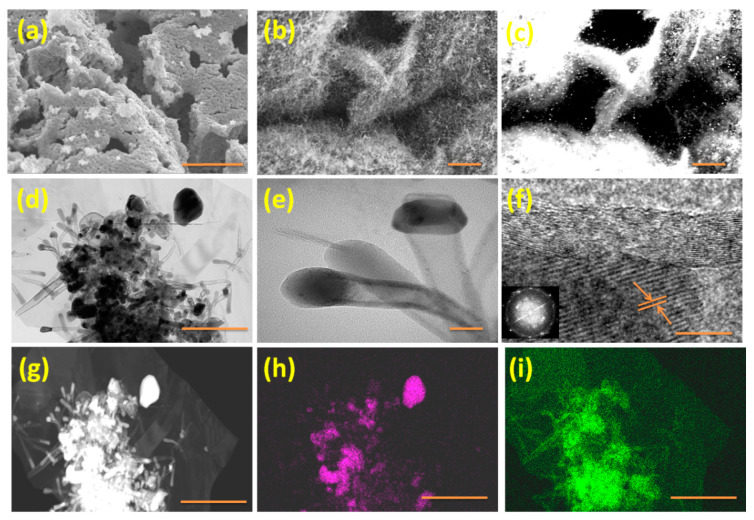
(**a**) Representative scanning electron micrograph (SEM) of DABCO Fe-MOF anchored on graphene oxide; (**b**) In-lens and (**c**) its corresponding secondary ion image of MDCNT@rGO; (**d**) Representative transmission electron micrograph (TEM)at low magnification; (**e**) High resolution transmission electron micrographs (HRTEM) of carbon nanotubes; (**f**) iron nanoparticles at the tip of CNT; (**g**) dark field TEM and its corresponding (**h**) iron and (**i**) nitrogen maps. Scale bars are 2 µm in (**a**–**c**); 500 nm in (**d**); 30 nm in (**e**); 10 nm in (**f**) and 500 nm in figures (**g**–**i**). Insert in (**f**) is the fast Fourier transform (FFT) pattern.

The morphological changes of Fe-MOF decorated graphene oxide and nitrogen-doped carbon nanotubes anchored on reduced graphene oxide (MDNCNT@rGO) were studied by SEM. Representative SEM micrographs of DABCO Fe-MOF nanoparticles anchored on graphene oxide exhibited in Figure 1a shows aggregation free and well-dispersed MOF nanoparticles distributed uniformly on the surface of graphene oxide substrate.

After being subjected to microwave radiation, the morphology of MDCNT@rGO (Figure 1b) shows ‘hairy structure’ with high density, micrometer long carbon nanotubes grown vertically on reduced graphene oxide. This is further evident from the corresponding ‘secondary electron’ image exhibited in Figure 1c. The reduced graphene oxide substrate and metal nanoparticles appear brighter compared to the carbon nanotubes due to their ability to backscatter more electrons. The mechanism of formation of CNTs by microwave pyrolysis of DABCO-based MOF can be explained by the following three concurrent steps. In the first step, when DABCO Fe-MOF decorated graphene oxide is subjected to microwave radiation, it experiences three types of effects: reflection, eddy current, and discharge effects. Of these three, the eddy current effect is the most prominent, wherein the MOF anchored on graphene substrate generate localized intense heat due to the adsorption of eddy currents leading to ionization and decomposition of the organic DABCO compound to nitrogenated hydrocarbon, which acts as the precursors for growth of carbon nanotubes resulting in the formation of three dime-sional, mesoporous MOF-derived CNT anchored on rGO substrate (MDCNT@rGO).

Detailed morphological studies were carried out with high-resolution TEM studies exhibited in Figure 1d,e, showing variable diameter and micrometer long-carbon nanotubes on rGO substrate. The maximum width of CNT was at the tip of. In contrast, the base was substantially narrower and is consistent with observations of O’Byrne et al. who reported a similar phenomenon in nitrogen-doped CNT synthesized from nitrogenated precursors [22,23]. HRTEM of the CNT at the tip exhibited in Figure 1f indicates presence of highly graphitized inner 22–24 walls, whereas the outermost 3–4 walls were comparatively less graphitized, disordered with interstitial defects, which can be attributed to the ‘knock-on effect’ of microwave radiation, which can damage and dislodge a carbon atom from the walls of CNT [24]. A dark-field TEM image of Figure 1d and its corresponding iron and nitrogen maps are shown in Figure 1g,h,i, respectively, wherein the metal nanostructures anchored on rGO and embedded inside the walls of CNT appear brighter, whereas the carbon moieties appear transparent. The iron map in Figure 1h shows iron nanoparticles are substantially present in CNT whereas the nitrogen map in Figure 1i shows higher concentration of nitrogen in CNT structures when compared to that rGO substrate. The presence of nitrogen in rGO substrate can be attributed to the reducing effect of DABCO. During microwave radiation, DABCO being a cyclic amine, disintegrates to ammonium compounds that react with the acidic hydroxyl, carbonyl, and carboxyl groups of graphene oxide to rGO [25].

**Figure 2:**
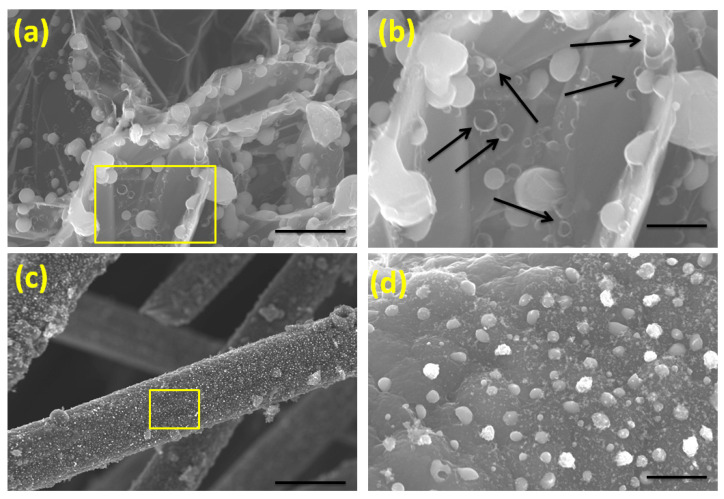
Morphology of carbon nanostructures obtained by pyrolysis of DABCO MOF-decorated graphene (**a**,**b**) and carbon fibers (**c**,**d**) in a traditional convection oven at 800 °C for 30 min. Scale bars are 2 µm, 500 nm, 10 µm, and 200 nm in (**a**–**d**), respectively.

To demonstrate the superiority of our newly developed microwave synthesis of 3DCNS from MOF precursor when compared to the widely practiced high-temperature calcination and pyrolysis, we heated DABCO Fe-MOF decorated graphene oxide and carbon fibers in a traditional convection oven at 800 °C for 30 min. The morphology of samples as studied by SEM is exhibited in Figure 2. On both substrates, no growth of CNTs was observed albeit in DABCO Fe-MOF decorated graphene oxide, presence of ‘hollow’ spherical carbon nanostructures as evident from Figure 2a,b can be seen, whereas in the case of carbon fiber substrates (Figure 2c,d), extensive localized etching of carbon fibers by metal nanoparticles occurred, which conclusively demonstrates that microwave radiation is the ‘rate-controlling step’ in the synthesis of CNT-based 3DCNS from MOF precursors.

**Figure 3:**
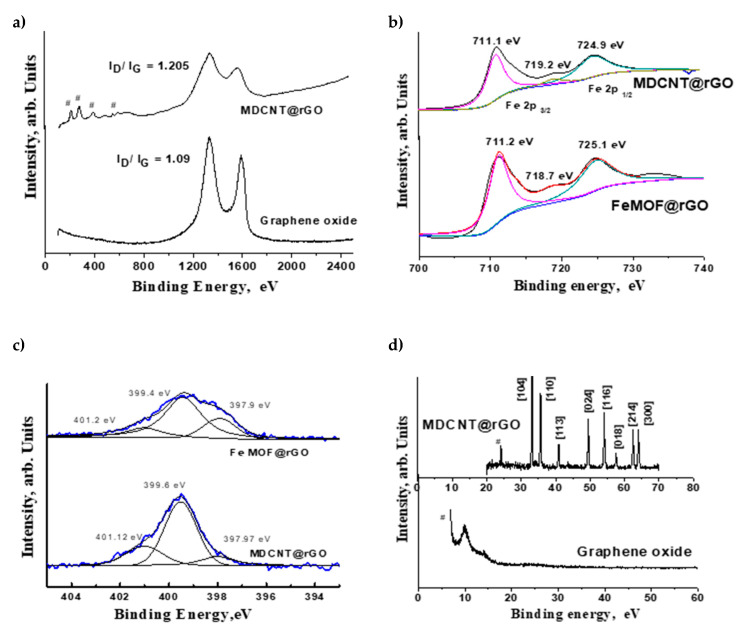
Raman spectra (**a**); Deconvoluted Fe2p (**b**); Deconvoluted N1 s XPS spectra (**c**) and X-ray diffraction (XRD) of MDCNT@rGO (**d**).

Raman spectroscopy, a powerful technique for the structural characterization of carbonaceous materials, has been used for the in-depth study of MOF-derived nitrogen-doped carbon nanotubes anchored on reduced graphene oxide (MDNCNT@rGO). Representative Raman spectra of graphene oxide plotted in Figure 3a shows the typical G band appearing at 1582 cm^−1^ and the disorder-related D band at about 1350 cm^−1^ [25], whereas in the case of and MDCNT@rGO, besides these two graphitic peaks, minor but visually discernible peaks at 220 and 492 cm^−1^; and 283, 396 and 596 cm^−1^ corresponding to A1g and Eg modes of iron moieties can also be observed [26]. Additionally, the I_D_/I_G_ ratio (ratio of intensities of D band to G band) from 1.09 in graphene oxide to 1.205 in MDCNT@rGO is observed, which indicates that the ‘in-plane’ defects on graphene oxide substrate generated during its synthesis by Tour’s method are repaired by the growth of carbon nanotubes.

The variation of electronic states of iron moieties before and after microwave-induced transformation of Fe MOF decorated GO to three-dimensional MDCNT@rGO was studied by X-ray photoelectron spectroscopy (XPS) measurements. The deconvoluted Fe2p core spectrum (Figure 3b) shows two broad peaks in the region 710 to 711 and 724 to 725 eV corresponding to Fe 2p_3/2_ and Fe 2p_1/2_, respectively. Generally, the spin-orbit splitting of the 2p bands is 13.4 eV, but in our case, it is 13.9 and 13.8 eV in FeMOF@rGO MDCNT@rGO, respectively. In the case of FeMOF@rGO, the Fe 2p_3/2_ peak centered at 711.23 eV can be deconvoluted into a single sharp rise at 711.2 eV corresponding to iron moieties bonded to metallo-organic complexes [27]. The peak at 725.1 eV corresponds to Fe ions embedded in a metal organic framework [28] with a minor satellite peak at 718.7 eV. However, in the case of MDCNT@rGO after deconvolution, the Fe 2p_3/2_ and Fe 2p_1/2_ peaks centered at 711.1 and 724.9 eV corresponds to iron nitride [29,30] and the minor satellite peak at 719.2 eV to the Fe ^3+^ oxidation state [31], which indicates that the iron moieties in MDCNT@rGO predominantly exists as iron nitrides with traces of oxide impurities. This can be further studied from the variation in the electronic state of nitrogen measured by XPS and plotted in Figure 3c, wherein deconvoluted XPS spectra of both FeMOF@rGO and MDCNT@rGO show three peaks. Still, the intensity and area under the peaks are greatly different. In MDCNT@rGO, the peak at 401.12 eV corresponds to graphitic nitrogen whereas this same peak in FeMOF@rGO shows a slight shift to 401.2 eV, which can be attributed to quartenary nitrogen. XPS survey scan exhibited in Figure S1 in supplementary file was used to calculate the elemental composition of MDCNT@rGO, which shows carbon: 79.27 oxygen: 6.03, nitrogen: 9.06 and iron: 13.49 wt %.

The peak at 399.4 and 399.6 eV in both the samples can be attributed to pyrrolic nitrogen, whereas the peak at 397.9 eV is pyridinic nitrogen. Figure 3d shows the typical XRD patterns of graphene oxide, iron decorated graphene, and G-Fe@NCNT composites. This is also reflected in the FTIR spectra of MDCNT@rGO exhibited as Figure S2 in supplementary file, which shows prominent peaks at 598, 1072, 1384, and 1637 cm^−1^ corresponding to Fe-C-N [32], C-N [33], physic-adsorbed nitrogen onto carbon [34] and C=N [35], respectively. The diffraction patterns of graphene oxide show a peak at 2θ of ~10 typical of graphene oxide synthesized by Hummer’s method while in the case of MDCNT@rGO, in addition, the carbon peak at ~24, very sharp peaks related to iron moieties at 2θ values of 33.21, 35.63, 40.88, 49.45, 54.07, 62.39, and 64.1 corresponding to (104), (110), (113), (024), (116), (214) and (300), reflecting the characteristics of iron nitride [36].

**Figure 4:**
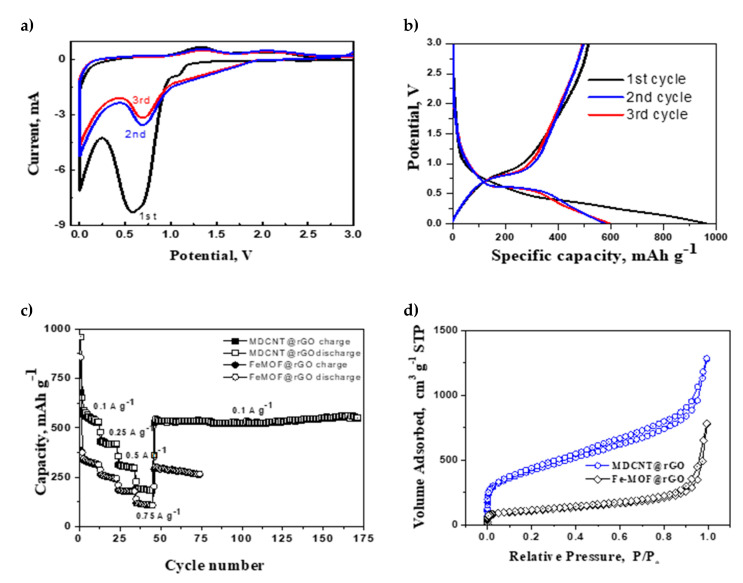
(**a**) Cyclic voltammetry studies, (**b**) galvanostatic discharge−charge cycling curves, (**c**) comparative cycling performance of MDCNT@rGO and FeMOF@rGO at various current densities, and (**d**) Brunauer-Emmett-Teller (BET) surface area of MDCNT@rGO and FeMOF@rGO.

The utility of our newly developed MDCNT@rGO derived from MOF precursors as potential anode material in sodium ion batteries is demonstrated below. Figure 4a depicts the voltage vs. capacity plots in the voltage range of 0.005−3.0 Vat current rate of 100 mA g^−1^ for first three cycles. In the first cathodic cycle, a sharp peak at around 0.59 V that can be attributed to the reduction (sodium insertion) and the reaction of iron ions with the electrolyte solution [37] can be observed, which in the subsequent cycles is shifted to the potential of 0.69 V. In the second and third cycles, the CV curves overlap with each other indicating good reversibility of the reduction process. In the anodic cycle, in all the three cycles shown in Figure 4a, a broad anodic peak at 1.32 V corresponding to the oxidation of Fe^0^ to Fe^3+^ (sodium extraction) was observed, indicating that the reversible and repeatable electrochemical reaction. Besides this broad peak, a minor hump at ~2.02 V in all the three reported cycles can be observed, which can be attributed to Na-ion insertion in the topological defects due to N-doping, which forms a disordered graphitic structure [38].

Figure 4b shows the charge–discharge profiles of MDCNT@rGO anodes at 100 mA g^−1^, exhibiting exceptional initial discharge and charge capacity of 513.3 and 957.4 mAh g^−1^, respectively with Coulombic efficiency of 53.61%. The extremely low Columbic efficiency in the first cycle is due to the solid electrolyte interface (SEI). Recent reports by Kang et al. [39] have shown that carbon electrodes in sodium ion batteries with carbonate-based electrolytes, tend to show very substantially lower Coulumbic efficiency, especially in the first cycle compared to glyme-based electrolytes. Despite low Coulumbic efficiency the charge capacity is high, which can be attributed to the synergetic effects of presence of alloying/dealloying type of electro-active iron oxide nanoparticles; adsorption of Na-ions on the surface of graphene and in the intra-tube mesopores between the vertically aligned nanotubes; adsorption of Na-ions on the extensive point defects on the walls of carbon nanotube walls due to nitrogen doping and finally, adsorption of sodium in the inter-laminar space in between the rGO substrates.

During the first discharge, the cycling profile showed two distinct regions consisting of visually discernible plateaus from 1.15 to 0.5 V and a progressive decrease from 0.5 V onwards, which corresponds to sodium insertion in iron moieties and in the nitrogen-doped moieties in CNT walls. Consequently, anodes based on our MDCNT@rGO show very high discharge capacities at all tested charge rates (Figure 3c) and, when tested for 125 cycles at a current density of 0.1A g^−1^, exhibits an exceptional capacity of 548 mAh g^−1^ and the observed values are almost double to that FeMOF@rGO-based electrodes. This shows that converting MOF into 3DCNS by simple additional step of microwave radiation for 45 s, the capacity of MOF-derived electrodes in sodium ion batteries can be almost doubled. This high value of capacity retention even after prolonged cycling can also be attributed to the substantial increase in the surface area as measured by N_2_-adsorption isotherms (Figure 4d), wherein MDCNT@rGO exhibited type I/II adsorption isotherm, with a surface area of 1285 m^2^ g^−1^ and a steady rise in the hysteresis loop in the P/P_0_ range of ≈0.44–0.94, which shows the formation of extensive micro/nanopores attributed to the ‘spacer’ functionality of nanotubes, which effectively inhibits the restacking of graphene due to van der Waals attraction. This spacer functionality of grown CNT provides effective pathways for diffusion of electrolytes and acts as highway for electron transport.

**Figure 5:**
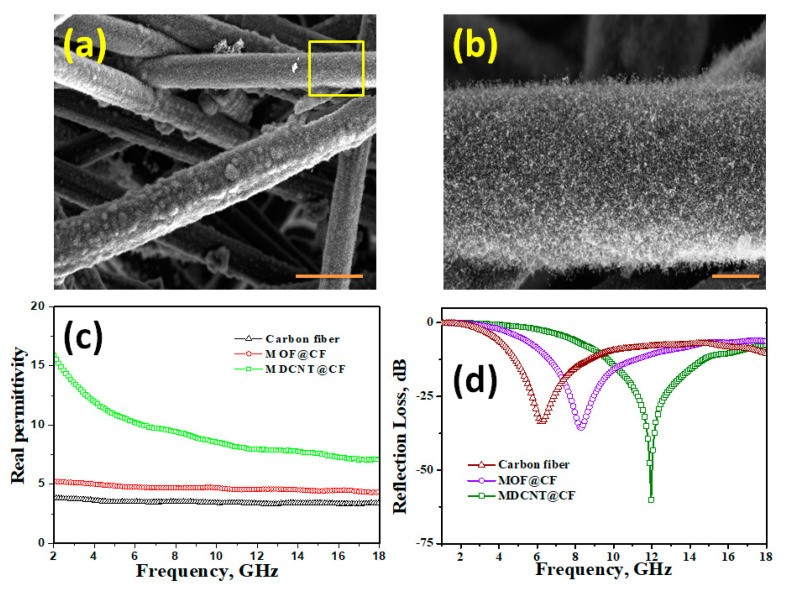
Representative SEM micrographs of MOF-derived CNT grown on carbon fiber (MDCNT@CF) (**a**). High-resolution image of highlighted portion (**b**), variation in real permittivity (**c**) and reflection loss (**d**) in electro-magnetic range. Scale bars in (**a**,**b**) are 3 µm and 1 µm, respectively.

We will also demonstrate the versatility of our newly developed MOF precursor to grow carbon nanotubes on carbon fibers. Pre-treated carbon fibers (10% HCl and DI water) were dipped in DABCO-Fe MOF methanolic solution 30 min, which followed by drying in a vacuum oven at 50 °C to evaporate methanol to obtain MOF-decorated carbon fibers, which were subsequently placed in a glass vial and microwave radiated for 30 s to obtain high-density CNTs vertically anchored on carbon fiber substrates (MDCNT@CF). SEM micrographs (Figure 5a,b) show high-density, micrometer-long CNT anchored all along the surface of carbon fibers, thereby unequivocally demonstrating that our newly developed DABCO-Fe MOF are excellent precursors for CNT on any microwave susceptible substrates. EMI is one of the growing invisible pollutions that can have adverse effects on human health. Generally, metals, especially magnetic nanostructures, were the original choice for developing high-performance microwave absorption and EM-shielding materials. Amongst various magnetic metals, iron-based nanostructures are especially worth mention because iron is cheap, malleable, abundant (4th commonest material in earth’s crust), a wide range of ionic states (from +2 to +7), the high saturation magnetization of 218 m^2^kg^−1^ at room temperature, etc. 

However, the higher density of metals has necessitated the need to develop lighweight EMI-absorbing materials. In this regard, carbon-based composites consisting of either graphene or CNT hybridized with metal nanoparticles are emerging as novel, lightweight EMI-absorbing materials. However, these novel forms of carbon hybrids are not strictly ‘structural’ materials. Structural materials can be defined as materials used or studied primarily for their mechanical properties, as opposed to their electronic, magnetic, chemical or optical characteristics. Amongst the emerging structural materials, carbon fibers have desirable properties like high stiffness, good tensile strength, low density, excellent chemical resistance etc. They are finding increasing utility in aerospace, civil engineering, military, and motorsports, etc. The aim of developing MDCNT@CF is to impart additional functionality of EMI shielding to a well-known structural material, like carbon fibers.

Figure 5c shows the variation in real permittivity in pristine carbon fibers, MOF-decorated carbon fibers and MDCNT. Amongst the three tested samples and in the whole frequency range of 2 to 18 GHz, the real permittivity of MDCNT@CF is substantially higher, especially in the lower frequency range, MDCNT@CF shows almost four times more permittivity when compared to pristine carbon fibers and MOF-decorated carbon fibers. In contrast, at the higher frequency range, the intensity of increase is about two times. The real permittivity is considered as the intrinsic polarization ability of a material arising due from the dipolar polarization and interfacial polarization at the microwave frequency [40]. In our case, the dipolar polarization is mainly due to the three-dimensional nanoporous carbon architectures of CNT on CF substrate. The interfacial polarization comes mainly from the interface of the cooperative consequence of the iron nanoparticle shell interfaces. This effect can also be observed in the variation of reflection loss vs frequency plot exhibited in Figure 5d wherein MDCNT@CF shows the highest loss at 12 GHz, which can be attributed to the three-dimensional structures consisting of high porosity and extensive surface area results in multi-interfaces. These accumulate the bound charges at the interfaces, causing the Maxwell–Wagner effect [41]. Both real permittivity and reflection loss data shows that our newly developed MDCNT@CF can act as excellent microwave shielding materials.

## 3. Experimental

### Synthesis of 3DCNS from MOF Precursors

The synthesis of 3DCNS from MOF precursors involved two steps [20] in which the first step was synthesis of MOF decorated graphene oxide. Tour’s method was used to synthesize 2 mg/mL methanolic dispersions of graphene oxide and to which 11.24 mmol of iron acetate and 5.52 mmol of DABCO were added. The mixture was ultrasonicated for 30 min and dried in a vacuum oven at 90 °C to remove methanol to yield solid Fe-MOF decorated graphene oxide. This powder was subsequently transferred into a glass vial and subjected to microwave radiation for 45 s at 700 W to yield a fluffy powdery solid of nitrogen-doped carbon nanotubes anchored on reduced graphene oxide (MDNCNT@rGO). Details of electrode preparation and characterization are provided in supplementary file, whereas EMI testing was carried out as per the protocol reported in our previous paper [20].

## 4. Conclusions

In summary, we have developed a fast and facile microwave method to synthesize mesoporous, 3D functional nanostructures consisting of high density, vertically anchored and nitrogen-doped CNT on reduced graphene substrates from DABCO MOF precursors. Morphological studies by SEM and TEM showed the developed 3D nanostructures are mesoporous with a surface area to the tune of 1285 m^2^ g^−1^. Raman spectra and chemical analysis by XPS indicated that the nitrogen moieties exist as a combination of pyridinic, pyrrolic, and graphitic nitrogen. When applied as anode in sodium ion batteries, the synthesized nanostructure exhibited exceptionally high storage capacity of 548 mAhg^−1^ even after 160 cycles emphasizing the ability of well-distributed iron nitride nanoparticles in three-dimensional nitrogen-doped CNT anchored rGO substrate to effectively accommodate the extreme volume changes occurring during sodiation/desodiation. The suitability of our newly developed technique to synthesize CNT on carbon fibers is also reported. The synthesized MDCNT@CF nanostructures show good performance for microwave shielding materials in the frequency range of 2 to 18 GHz.

## Data Availability

Data is contained within the article or supplementary material.

## Supplementary Materials

The following are available online, Table S1 Representative morphologies of MOF derived carbons; Materials and Methods; Electro-chemical tests; Figure S1: XPS survey scan of MDCNT@rGO; Figure S2. FTIR spectra of MDCNT@rGO indicating presence of nitrogen moieties; Figure S3: XRD of Fe-MOF synthesized on carbon fiber substrate; Figure S4: Representative SEM morphology of DABCO MOF decorated carbon fibers; Figure S5: Secondary electron image corresponding to MDCNT@CF shown in Figure 5a,b of the manuscript.

## Sample Availability

Samples of the compounds used are available from the authors.
