# Supplementary material for: Metal Organic Frameworks Derived Fe-N-C Nanostructures as High-Performance Electrodes for Sodium Ion Batteries and Electromagnetic Interference (EMI) Shielding
Source: Molecules. 2021 Feb 15;26(4):1018. doi: 10.3390/molecules26041018 (PMC7919031; doi:10.3390/molecules26041018)
Supplement: Supplementary file 1 [file molecules-26-01018-s001.pdf]

Table S1 Representative morphologies of MOF derived carbons

S. No	Material	Carbonization Temperature/Time/atmosphere	Morphology	Reference
1	Electrospun Zn-MOF	1000C/6 hours/Argon	Carbon fiber web	1
2	MIL-101, Alumina template	600 to 1000 C/ 5 hours/ Argon	Honeycomb carbons	2
3	Zn MOF-74	600 to 1000 C/ 5 hours/ Argon	Carbon nanorods	3
4	Co-MOF	500 C/ 2 hours/ Nitrogen	Disordered carbons	4
5	Basolite, F300	700 to 900 C/ 5 hours/ Argon	hollow carbon nanospheres	5
6	metal @ZIF-8	600 to 900 C/ 4 hours/ H_2 and Argon	Porous carbons	6
7	Cobalt-Melamine-BDC	700 to 900 C/ 4 hours/ Nitrogen	Disordered CNT carbons	7
8	Cobalt-triazine networks	700 C/ 3 hours/ Vacuum	Porous polyhedrons	8
9	Fe-MIL	500 C/ 2 hours in Argon; subsequent annealing in air for 2 more hours	Iron oxide@C	9
10	Ni-MOF	500 C/2 hours/Nitrogen	Nickel rich hollow carbons	10
11	ZIF-67	800 to 1000 C/ 5 hours/ Nitrogen	Sodalite structured mesoporous carbons	11
12	Ti-amino BDC	1000 C/8 hours/Argon	3D carbon cuboids	12
13	ZIF-67/LDH	400 C/ 3.3 hours/ Air	Hollow nanocages	13
14	ZIF-8	600 and 1000C/ 1 hour/ Nitrogen	Mesoporous carbons	14
15	ZIF-67	350 C/ 2 hours/ Nitrogen	Cobalt rich carbon Hollow prisms	15
16	ZIF-8/MnO_2 Nanorods	700 C/ 4 hours/ Argon	$ZnMnO_4$ carbon rods	16
xx	DABCO based MOF	Microwave/ 45 seconds/ Air	NCNT on rGO/ NCNT on Carbon fiber	

Materials and Methods

Materials:

99.5% pure graphite (grade-QSG) was purchased from Samjung (C & G, Korea) whereas reagent grade sulphuric acid (H_2SO_4), hydrochloric acid (HCl), sodium nitrate ($NaNO_3$), hydrogen peroxide, potassium permanganate ($KMnO_4$), 1,4-diazabicyclo[2.2.2]octane (DABCO) and iron(III) acetatewere purchased from Sigma-Aldrich, Korea, and were used as received. Microwave irradiation was carried out in a domestic microwave oven manufactured by Daewoo Korea, Model number: KR-B202WL with output power of 700 W operating at 2450 MHz.

Methods:

Field-emission scanning electron microscopy (FE-SEM, Nova NanoSEM 230 FEI operating at 10kV and TALOS F200X Transmission electron microscopy operating at 200 kV were used to study SEM and HRTEM morphology, respectively. Energy-dispersive X-ray spectroscopy (EDS) scans were taken on TALOS F200x (FEI) using the in-built UltraFast mapping SDD-EDS using Mn-Kα detector. Due to inherently excellent electrical conductivity of all the synthesized 3-D carbonaceous materials, viz. Fe@NCNT-rGO, Co@NCNT-rGO and Fe@NCNT-CF, there was no necessity of metal coating for SEM testing. Structural properties were studied by X-ray diffraction and Raman spectroscopy using Rigaku D/max-2550V, Cu-Kα radiation and LabRAM HR evolution UV/vis/NIR spectrometer. High resolution X-ray photoelectron spectroscopy studies were carried out on a Sigma Probe Thermo VG spectrometer using Mg Kα X-ray sources. The XPS spectra were curve fitted with a mixed Gaussian-Lorentzian shape using XPSPEAK version 4.1. BET (Brunauer-Emmett-Teller) surface area was measured by Nitrogen adsorption and desorption isotherms at 77K using a BEL Japan Inc. Belsorp Mini II Surface Area. Prior to testing, the sample was first degassed for 24 h at 250 °C.

Electro-chemical tests

All the electrochemical measurements were conducted on a CHI 660 electrochemical station (CH Instruments, Inc.) with a conventional three-electrode system. In a typical electrochemical measurement, a total of 5 mg of catalysts was dispersed by sonication in a mixture of 480 μL isopropanol (J&K Scientific LTD.) and 20 μL of Nafion aqueous solution (5 wt.%, DuPont) for 30 min. 12 μL of the above suspension was gently dropped onto a glassy carbon rotating disk electrode (RDE, 5 mm diameter) or rotating ring-disk electrode (RRDE, 4.93 mm inner diameter and 5.38 mm outer diameter) and dried naturally in air. The above procedure was repeated twice which results in a total loading mass of about 0.6 mg/cm2. The electrolyte was 0.1 M KOH aqueous solution, and the reference and counter electrodes were saturated calomel electrode (SCE) and Pt wire, respectively. The electrolyte was saturated with O2 before the experiment, and O2 was continuously supplied during the experimental operation. All potential values reported in this study were converted to the reversible hydrogen electrode (RHE) scale, according to the equation: $E_{RHE} = E_{SCE} + 0.0591pH + 0.242$. For the stability test, the working electrode ran at - 0.7 V vs SCE for 60,000 s in O2-saturated 0.1M KOH with a rotation rate of 1600 rpm. For comparison, commercial Pt/C (20 wt.% Pt) powder was tested under the same conditions, with a loading mass of 0.25 mg cm^{-2}. Linear sweep voltammetry (LSV) was measured by RDE/RRDE technique the with the scan rate of 10 mV/s at various rotating speeds from 400 to 2400 rpm.”

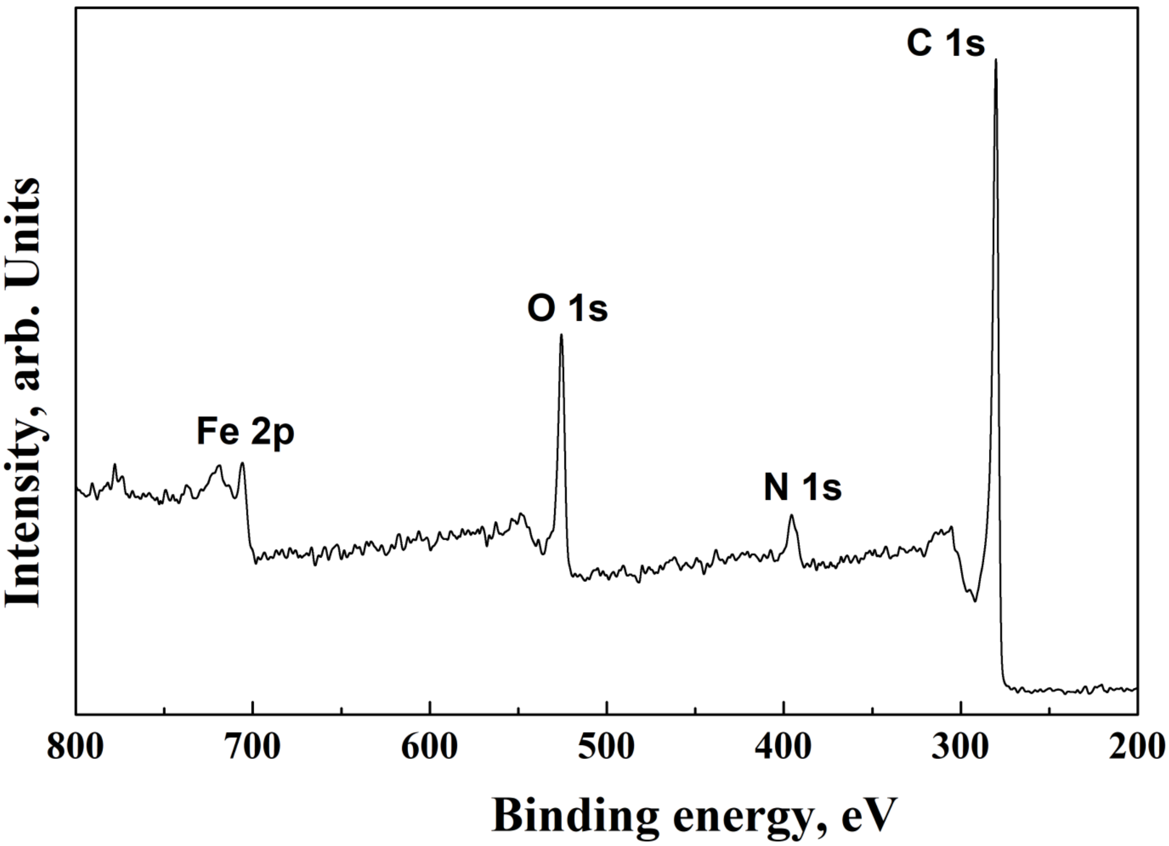

Figure S1: XPS survey scan of MDCNT@rGO.

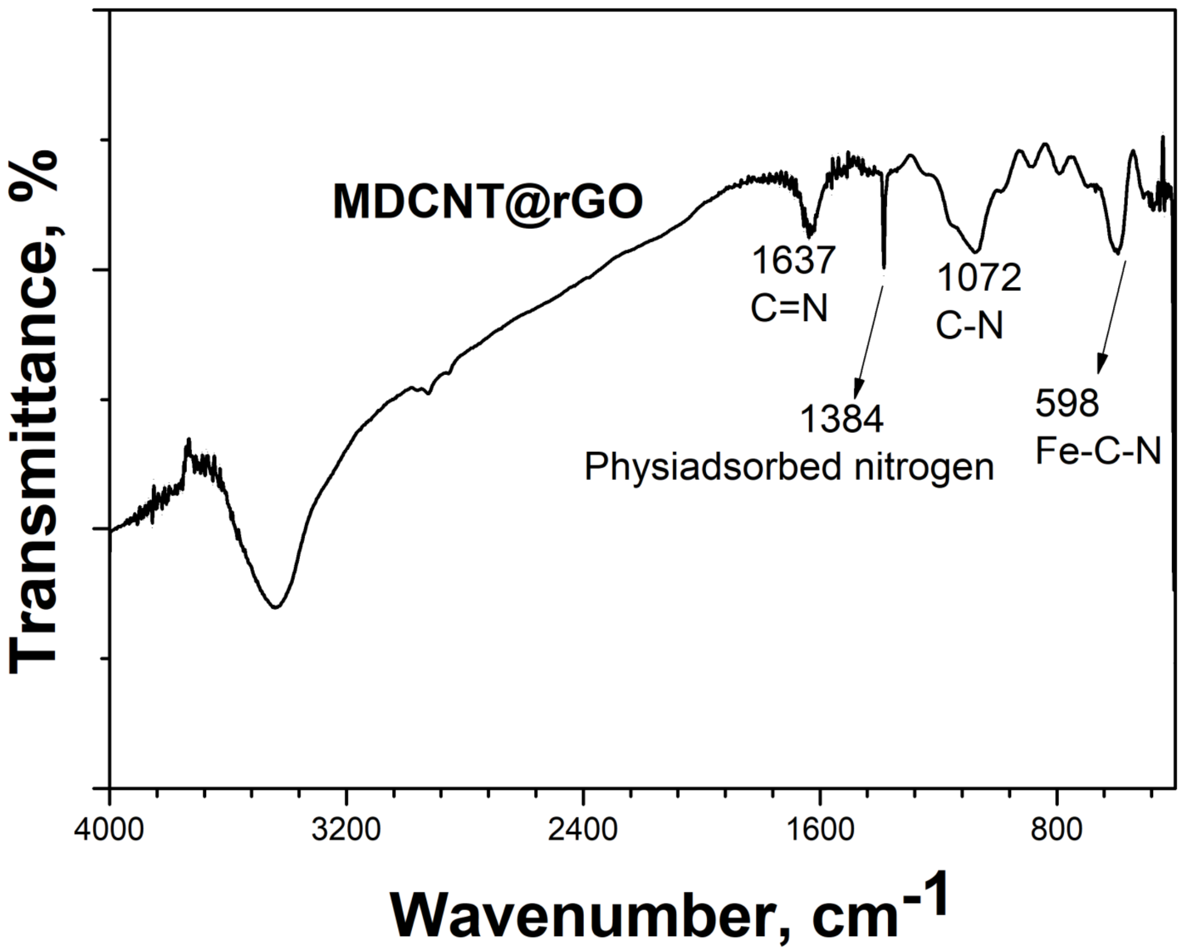

Figure S2. FTIR spectra of MDCNT@rGO indicating presence of nitrogen moieties

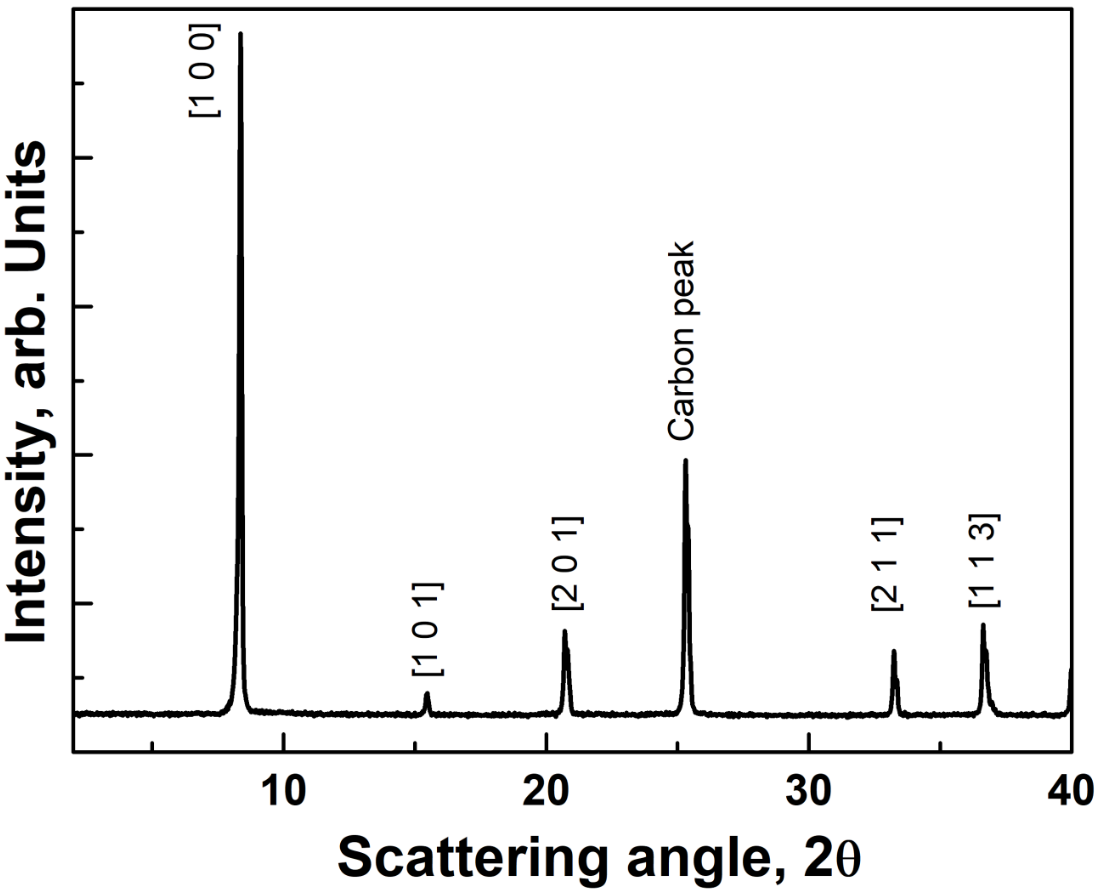

Figure S3: XRD of Fe-MOF synthesized on carbon fiber substrate.

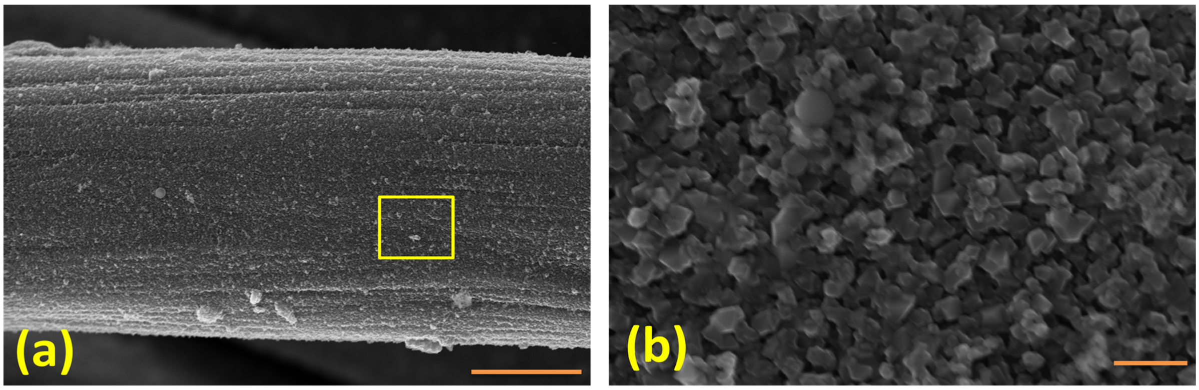

Figure S4 Representative SEM morphology of DABCO MOF decorated carbon fibers. Scale bars are 2 μm and 200 nm in (a) and (b), respectively.

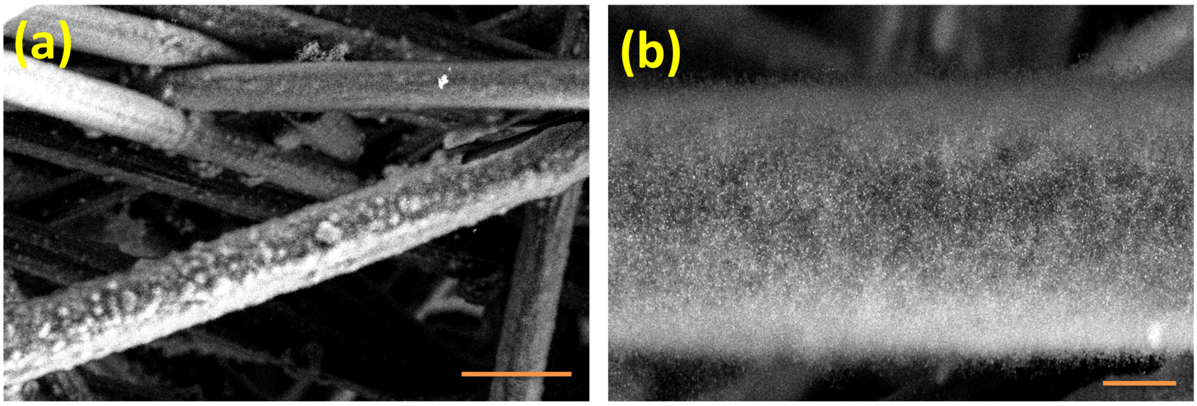

Figure S5 Secondary electron image corresponding to MDCNT@CF shown in Fig. 5(a) and 5(b) of the manuscript. Scale bars are 3μm and 1μm respectively.

References

1. I. T. Kim, S. Shin, M. W. Shin. Development of 3D interconnected carbon materials derived from Zn-MOF-74@carbon nanofiber web as an efficient metal-free electrocatalyst for oxygen reduction. Carbon, 2018, 135, 35–43.

2. Q.-L. Zhu, W. Xia, T. Akita, R. Zou, Q. Xu. Metal-Organic Framework-Derived Honeycomb-Like Open Porous Nanostructures as Precious-Metal-Free Catalysts for Highly Efficient Oxygen Electroreduction. Adv Mater., 2016, 28 (30), 6391–6398.

3. P. Pachfule, D. Shinde, M. Majumder, Q. Xu. Fabrication of carbon nanorods and graphene nanoribbons from a metal–organic framework. Nat. Chem., 2016, 8 (7); 718–724.

4. B. Lin, A. Wang, Y. Guo, Y. Ding, Y. Guo, L. Wang, W. Zhan, F. Gao. Ambient Temperature NO Adsorber Derived from Pyrolysis of Co-MOF(ZIF-67)). ACS Omega, 2019, 4 (5); 9542–9551.

5. M. Klose, R. Reinhold, K. Pinkert, M. Uhlemann, F. Wolke, J. Balach, T. Jaumann, U. Stoeck, J. Eckert L. Giebeler. Hierarchically nanostructured hollow carbon nanospheres for ultra-fast and long-life energy storage. Carbon, 2016, 106, 306–313.

6. Z. Qi, Y. Pei, T. W. Goh, Z. Wang, X. Li, M. Lowe, R. V. Maligal-Ganesh, W. Huang. Conversion of confined metal@ZIF-8 structures to intermetallic nanoparticles supported on nitrogen-doped carbon for electrocatalysis. Nano Research, 2018, 11 (6); 3469–3479.

7. H. Zhong, Y. Luo, S. He, P. Tang, D. Li, N. Alonso-Vante, Y. Feng. Electrocatalytic Cobalt Nanoparticles Interacting with Nitrogen-Doped Carbon Nanotube in Situ Generated from a Metal–Organic Framework for the Oxygen Reduction Reaction. ACS Appl. Mater. Interfaces., 2017, 9 (3); 2541–2549.

8. J.-D. Yi, R. Xu, G.-L. Chai, T. Zhang, K. Zang, B. Nan, H. Lin, Y.-L. Liang, J. Lv, J. Luo, R. Si, Y.-B. Huang, R. Cao. Cobalt single-atoms anchored on porphyrinic triazine-based frameworks as

bifunctional electrocatalysts for oxygen reduction and hydrogen evolution reactions. J. Mater. Chem. A, 2019,7 (3), 1252-1259.

9. K. Wang, M. Chen, Z. He, L. Huang, S. Zhu, S. Pei, J. Guo, H. Shao, J. Wang. Hierarchical Fe3O4@C nanospheres derived from Fe2O3/MIL-100(Fe) with superior high-rate lithium storage performance. J. Alloys Compd. ,2018 ,755 ,154–162.

10. X. Lin, S. Wang, W. Tu, Z. Hu, Z. Ding, Y. Hou, R. Xu, W. Dai. MOF-derived hierarchical hollow spheres composed of carbon-confined Ni nanoparticles for efficient CO_2 methanation. Catal. Sci. Technol., 2019, 9(3); 731–738.

11. N. L. Torad, R. R. Salunkhe, Y. Li, H. Hamoudi, M. Imura, Y. Sakka, C.-C. Hu, Y. Yamauchi. Electric Double-Layer Capacitors Based on Highly Graphitized Nanoporous Carbons Derived from ZIF-67. Chem.: Eur. J., 2014, 20 (26); 7895–7900.

12. A. Banerjee, K. K. Upadhyay, D. Puthusseri, V. Aravindan, S. Madhavi, S. Ogale. MOF-derived crumpled-sheet-assembled perforated carbon cuboids as highly effective cathode active materials for ultra-high energy density Li-ion hybrid electrochemical capacitors (Li-HECs). Nanoscale, 2014, 6 (8), 4387-4384.

13. W. Kong, J. Li, Y. Chen, Y. Ren, Y. Guo, S. Niu, Y. Yang. ZIF-67-derived hollow nanocages with layered double oxides shell as high-Efficiency catalysts for CO oxidation. Appl. Surf. Sci. , 2018, 437, 161–168.

14. F. J. Martín-Jimeno, F. Suárez-García, J. I. Paredes, M. Enterría, M. F. R. Pereira, J. I. Martins, J. L. Figueiredo, A. Martínez-Alonso, J. M. D. Tascón. A ‘Nanopore Lithography’ Strategy for Synthesizing Hierarchically Micro/Mesoporous Carbons from ZIF-8/Graphene Oxide Hybrids for Electrochemical Energy Storage. ACS Appl. Mater. Interfaces, 2017, 9 (51), 44740–44755.

15. L. Yu, J. F. Yang, X. W. D. Lou. Formation of CoS2 Nanobubble Hollow Prisms for Highly Reversible Lithium Storage. Angew. Chem. Int. Ed., 2016, 55 (43); 13422–13426.

16. M. Zhong, D. Yang, C. Xie, Z. Zhang, Z. Zhou, X.-H. Bu. Yolk-Shell $MnO@ZnMn_2O_4$/N-C Nanorods Derived from α-MnO_2/ZIF-8 as Anode Materials for Lithium Ion Batteries. Small, 2016, 12 (40); 5564–5571.

17. This work.
